# Supplementary material for: Trajectories and correlates of opioid prescription receipt among patients experiencing interpersonal violence
Source: PLoS One. 2022 Sep 9;17(9):e0273846. doi: 10.1371/journal.pone.0273846 (PMC9462725; doi:10.1371/journal.pone.0273846)
Supplement: S1 Table — (DOCX) [file pone.0273846.s001.docx]

**Supplementary Materials**

**International Classification of Diseases 9^th^ and 10^th^ edition (ICD-9, ICD-10) codes used to define model covariates**

| **Covariate** | **ICD-10 Code** | **ICD-9 Code** |
| --- | --- | --- |
| *Chronic Pain* | | |
| Chronic Pain, not elsewhere classified | G89.2 | 338.2 |
| Chronic pain syndrome | G89.4 | 338.4 |
| Fibromyalgia | M79.7 | 729.1 |
| Irritable Bowel Syndrome | K58.0 | 564.1 |
| Interstitial cystitis/Bladder pain syndrome | N30.1, N30.2, N30.3 | 595.1, 595.2, 595.3 |
| Migraine | G43 (exclude G43.6, G43.A) | 346 (exclude 346.6) |
| Chronic low back pain | M54.5, M54.4, M54.89 | 724.2 |
| Chronic Fatigue Syndrome | R53.82 | 780.71 |
| Endometriosis | N80 | 617 |
| *Substance Use Disorders* | | |
| Alcohol related disorders | F10 | 303, 305.0 |
| Opioid related disorders | F11 | 304.0, 304.7, 305.5 |
| Cannabis related disorders | F12 | 304.3, 305.2 |
| Sedative, hypnotic, or anxiolytic related disorders | F13 | 304.1, 305.4 |
| Cocaine related disorders | F14 | 304.2, 305.6 |
| Other psychoactive substance related disorders | F19 | N/A |
| Unspecified/Other drug dependence | N/A | 304.6, 304.8, 304.9, 305.8, 305.9 |
| *Mental Health* | | |
| Depression | F31.3, F31.4, F31.5, F32, F33, F34.1, F41.2, F43.2 | 296.2, 296.3, 296.5, 300.4, 309, 311 |
| Schizophrenia, schizotypal, delusional, and other non-mood psychotic disorders | F20-F29 | 295, 297, 298.1, 298.3, 298.4, 298.8, 298.9 |
| Anxiety, dissociative, stress-related, somatoform and other non-psychotic mental disorders | F40-F48 (exclude F41.2, F43.2) | 298.2, 300 (exclude 300.4) |
| Disorders of adult personality and behavior | F60-F69 | 301 |
| Other mood disorders | F30, F31.1, F31.2, F31.6, F31.7, F31.8, F31.9, F34.0, F34.8, F34.9, F35-F39 | 296.0, 296.1, 296.4, 296.6, 296.7, 296.8, 296.9 |
