# Supplementary material for: Trajectories and correlates of opioid prescription receipt among patients experiencing interpersonal violence
Source: PLoS One. 2022 Sep 9;17(9):e0273846. doi: 10.1371/journal.pone.0273846 (PMC9462725; doi:10.1371/journal.pone.0273846)
Supplement: S2 Table — (DOCX) [file pone.0273846.s002.docx]

**Supplementary Materials**

**Summary of Key Characteristics for Each Opioid Prescription Trajectory Class**

| Trajectory Class | Summary of Key Characteristics |
| --- | --- |
| Never | Youngest; similar proportion of males and females to classes 5 and 6; highest proportion of Hispanic; highest proportion of never smokers; lowest prevalence across most chronic pain, alcohol and drug related disorders, and mental health disorders; lowest Charlson Comorbidity Index |
| Class 1: Start medium, end medium, moderate decrease | Highest proportion of males; highest proportion of insured, similar to class 2; highest proportion of current smokers, similar to class 3; highest prevalence for irritable bowel syndrome and migraine; highest prevalence of alcohol related disorders, cannabis, and unspecified/other drug dependence; highest prevalence of disorders of adult personality and behavior |
| Class 2: Start high, end high, slight increase | Oldest; similar proportion of males and females to class 4; highest proportion of White, non-Hispanic; highest proportion of varying insurance status; similar illicit drug use history to class 4; highest prevalence across most chronic pain conditions, including: chronic pain, not elsewhere classified, chronic low back pain, chronic pain syndrome, fibromyalgia, interstitial cystitis/bladder pain; highest prevalence cocaine and other psychoactive substance disorders; highest prevalence of depression and other mood disorders; highest Charlson Comorbidity Index |
| Class 3: Start medium, end low, sharp decrease | Similar in age to classes 4 and 6; highest proportion of females; lowest illicit drug use history |
| Class 4: Start low, end high, sharp increase | Highest proportion of past smokers; highest prevalence for chronic fatigue syndrome and endometriosis; highest prevalence of opioid and sedative/hypnotic/anxiolytic disorders; highest prevalence of schizophrenia/schizotypal/delusional/other non-mood psychotic disorders and anxiety/dissociative/stress-related/somatoform/other non-psychotic mental disorders |
| Class 5: Start low, end medium, moderate increase | Highest proportion of Black, non-Hispanic; highest illicit drug use history |
| Class 6: Start low, end low, stable | Highest proportion of non-insured; low prevalence of chronic pain, alcohol and drug related disorders, and mental health disorders |
