# Supplementary material for: Trajectories and correlates of opioid prescription receipt among patients experiencing interpersonal violence
Source: PLoS One. 2022 Sep 9;17(9):e0273846. doi: 10.1371/journal.pone.0273846 (PMC9462725; doi:10.1371/journal.pone.0273846)
Supplement: Attachment — Submitted filename: IPRC Opioid Trajectory Response to Reviewers.docx [file pone.0273846.s003.docx]

PONE-D-22-04197
Trajectories and correlates of opioid prescription receipt among patients experiencing interpersonal violence
*PLOS One*

| **Reviewer #1** | **Response** |
| --- | --- |
| Not distinguishing some indication of high-risk opioid prescribing (e.g., long-term or high dose) from any prescription opioid receipt seems like an important limitation, given that the introduction frames potential adverse outcomes (nonmedical use, OUD) in terms of prevalence among patients receiving long-term opioid therapy. The authors note that dosage and duration information is not readily available prior to April 2014 – might it be possible to analyze a subset of the data for which these measures are available to gain some insight into patterns of high-risk prescriptions (perhaps just looking at characteristics associated with high-risk prescriptions, if there is not enough longitudinal data to examine trajectories over time)? | We appreciate Reviewer 1’s recommendation to gain insight into patterns of high-risk opioid prescription. Unfortunately, the data available (even after 2014) is not sufficient to categorize prescriptions as high-risk. The biggest limitation for this is the lack of days’ supply variable in the EMR system. This variable is a free text field, and one group that has been analyzing this data has found >7000 different variations in the text strings which limits calculations of daily MMEs as well. We have added additional text to highlight that post-baseline OUD diagnoses were highest among trajectory groups with highest likelihood of opioid prescription over time. This provides some preliminary evidence that these are high-risk trajectory groups. We expand on the implications of this in the discussion along with recommendations for future research. |
| Related to the above point, the introduction could make a better case for why it is useful to examine trajectories of any prescription opioid receipt in this population (e.g., are there data demonstrating increased risk for adverse outcomes resulting from any opioid exposure in this population)? The section of the introduction stating “one population particularly vulnerable to deleterious effects of opioid use is individuals with a history of interpersonal violence” could be expanded in this regard. | Additional information has been added to the introduction highlighting the increased risk for OUD and other adverse health outcomes among those who have been prescribed opioids, both in the general population and among those with a history of IPV. |
| Additionally, the need for research examining high-risk opioid prescriptions in this population deserves more attention in the limitations section. | Thanks for noting this. We have now added more discussion to this section, to clarify the importance of MMEs and the implications due to the lack of MMEs on our study implications. |
| Please explain in the methods why baseline was defined differently for patients who had ≥1 opioid prescription (date of first prescription) and patients who had none (date of first EHR entry), rather than defining consistently as the date of first EHR entry during the study period for all patients. | For the trajectory analysis (Aim 1), baseline was defined as date of first opioid prescription. Those who did not receive an opioid prescription during the study period were not included in the trajectory analysis but rather placed in a “Never” class. So, technically the Never group does not have a baseline for Aim 1 analyses. We see how our description is confusing and revised this text in the Design and Analysis section to clarify. |
| It is unclear to me in the methods whether referral for interpersonal violence services preceded the measured trajectory of opioid receipt. Did the referral always precede the baseline date (first opioid prescription or EHR entry during the study period), or could patients have entered the cohort prior to being referred? | Given that experiences of interpersonal violence are often chronic rather than an isolated event, referral for violence related services is not an accurate indicator of the “beginning” of violence. Thus, we did not limit data extraction based on date of referral to the Beacon Program, but rather extracted relevant variables across the study period (January 2004 – August 2019). We have included additional text in the Variables section to clarify. |
| The variables section of the methods should more clearly state that the outcome was defined as binary indicator of any prescription opioid receipt in each month. | We have added additional information to the description of the prescription opioid receipt variable clarifying that this outcome was defined as a binary indicator of any prescription opioid receipt for each month of the study period. |
| Please provide more information about how smoking and illicit drug use history were measured – are these routinely screened for and documented in this health care setting? | We have clarified that social history information is routinely screened for by providers within the UNC Health System and documented in each patient’s EHR. |
| The implications highlighted in the discussion do not clearly follow from these findings. For example, the authors argue that history of interpersonal violence should be considered when assessing for opioid risk and in pain management care – this is presumably because people with a history of interpersonal violence are at higher risk for adverse opioid-related outcomes than those without such a history, however that was not examined in this study. What are the implications of findings from the present study—which examined trajectories of any prescription opioid receipt and associated baseline characteristics among people with history of interpersonal violence—for clinical care? | We have revised the discussion section to better align with our study findings. This included removing reference to opioid risk tools and focusing on increased monitoring for co-morbid conditions during pain management. |
| Abstract, line 15: I suggest clarifying here that classes are characterized by “probability of any prescription opioid receipt at the start and end of the study period” and “change in probability over time” | We have included these additional details in the abstract. |
| Introduction, line 23: I think this is meant to say “opioid use disorder” instead of “opioid disorder” | Revised |
| Introduction, lines 41-43: Please clarify whether interpersonal violence is associated with increased likelihood of these outcomes. | We have clarified that IPV is associated with an increased likelihood of prescription opioid use, non-medical use of opioids, and heroin use. |
| **Reviewer #2** |  |
| Were models looking at frequency or number of opioid prescriptions, or at something else? At what time intervals? It is not clear to me exactly what “high, medium, low, etc.” means here, which limits the ability to interpret the clinical relevance of the findings. | The models examined prescription opioid receipt as a binary indicator (yes/no) for each month of the study period. The trajectories were modeled to estimate the probability of prescription opioid receipt within each class over time (e.g., high, medium, low). We have added additional text to the measures section to clarify how we defined this outcome variable. |
| I believe the paper would be stronger if the authors could more directly explain how their findings add new understanding that will inform clinical practice. It is notable that such a high proportion of this cohort with histories of IPV had received an opioid prescription, that mental health and substance -related diagnoses were so prevalent, and that these comorbidities were (not surprisingly) more prevalent among classes with higher probability of receiving opioid prescriptions. What do we take away from this? I appreciate the discussion about synergism and the need for better screening tools/practices. Perhaps one implication is that when treating people with chronic pain and IPV, it may be particularly important to monitor closely and to offer treatment that is trauma-informed, destigmatizing and integrated, so that synergistic comorbidities are addressed. | We have revised the discussion section to emphasize the need for improved monitoring of co-morbid conditions during pain management and the need to provide trauma-informed pain management. |
| It seems all data was electronically abstracted from the EHR, but I do not see that this is made explicit. Please clarify. | Yes, all data were electronically abstracted from the EHR. We have added additional text in the Variables section to clarify. |
| Would be helpful to characterize the cohort by type of IPV–in particular, how many were older /vulnerable adults vs people who had experienced intimate partner violence/sexual trauma. | Unfortunately, the records we obtained from the Beacon program do not provide this distinction so we are unable to characterize the cohort by type of IPV. |
| In Table 1, under the class analysis, the percentages are based on the total cohort of 1587. I wonder if these should be calculated based on the number of participants who received an opioid rx and were included in the class analysis. | We included percentages for the total cohort in Table 1 to provide demographics/participant characteristics for the entire sample rather than having a separate sample characteristics table. |
| Line 160: the text states that 70.8% of participants were in the never + class 6 groups; however, the percentages in those groups do not sum to 70%. This may need correction or clarification. | Thank you for identifying this error. We have corrected this value. |
| The lack of dose/MED is a significant limitation. While noted in the limitations section, I wonder if the authors could discuss this further. What specifically is the clinical relevance of the classes that were identified by their model, and how does the lack of MED data affect the clinical implications? | Thanks for noting this. We have now added more discussion to this section, including the potential clinical relevance of our study and how lack of MMEs affects our study implications. |
| Does the calculation of standardized differences allow for calculations of significance and for adjustment for confounders? I am not sure what to make of differences in prevalence alone. Especially regarding gender differences, as the numbers were small. | The standardized difference is a measure of effect size and provides information about the similarity or dissimilarity of two groups with respect to specific covariates. These measures are independent of sample size and thus are not interpreted based on calculations of significance, but rather the size of the effect (e.g., Cohen’s effect size indices of 0.2, 0.5, and 0.8 represent small, medium, and large effect sizes, respectively).  Given the number of covariates examined, we felt providing a visual presentation of the standardized differences in Fig. 2 allowed for a better summary of overall characteristics for each class. |
| Line 217: prescription opioid users could be rephrased to more patient-centered language (“people who use prescription opioids”) | Agreed and thank you. We have made this revision. |
| How do the authors interpret the prevalence of opioid related diagnoses in the different classes, given that people with OUD at baseline were excluded: does this suggest that these patients developed OUD or simply reflect better capture of diagnoses, or both? | Yes, the OUD prevalence, at least in part, indicates individuals who were diagnosed with OUD after opioid prescription. While we are unable to determine for certain if these rates are a result of new OUD cases or increased monitoring, these results do provide some preliminary evidence that IPV may increase risk for the development of OUD among those prescribed opioids. We have highlighted these findings in the results and discussion sections. |
| Line 258: Given that the data does not include any development of OUD or other outcomes, I’m not sure the findings justify this statement. | We have deleted this statement and revised the discussion section to more clearly align with our study findings. |
| Line 259: Please change "abuse" to "use" | Revised |
| I believe the conclusions (final paragraph) are overstated and recommend that they be reworded. Without dose information, I’m not sure this can be considered “comprehensive.” And I’m not convinced that the authors have described how “understanding profiles of risk informs clinical screening protocols and precision of clinical treatment plans…” I think the key questions are how these risk profiles translate into improving appropriate / safety of opioid prescribing and chronic pain management in general for this population. | We have revised the conclusions so they are not overstated and align better with our study findings. |
